# A fast and tuneable auxin‐inducible degron for depletion of target proteins in budding yeast

**DOI:** 10.1002/yea.3362

**Published:** 2018-11-12

**Authors:** Gonzalo I. Mendoza‐Ochoa, J. David Barrass, Barbara R. Terlouw, Isabella E. Maudlin, Susana de Lucas, Emanuela Sani, Vahid Aslanzadeh, Jane A.E. Reid, Jean D. Beggs

**Affiliations:** ^1^ Wellcome Centre for Cell Biology, School of Biological Sciences University of Edinburgh Edinburgh UK

**Keywords:** auxin, degron, estradiol, protein depletion, regulated gene expression, yeast

## Abstract

The auxin‐inducible degron (AID) is a useful technique to rapidly deplete proteins of interest in nonplant eukaryotes. Depletion is achieved by addition of the plant hormone auxin to the cell culture, which allows the auxin‐binding receptor, TIR1, to target the AID‐tagged protein for degradation by the proteasome. Fast depletion of the target protein requires good expression of TIR1 protein, but as we show here, high levels of TIR1 may cause uncontrolled depletion of the target protein in the absence of auxin. To enable conditional expression of TIR1 to a high level when required, we regulated the expression of TIR1 using the β‐estradiol expression system. This is a fast‐acting gene induction system that does not cause secondary effects on yeast cell metabolism. We demonstrate that combining the AID and β‐estradiol systems results in a tightly controlled and fast auxin‐induced depletion of nuclear target proteins. Moreover, we show that depletion rate can be tuned by modulating the duration of β‐estradiol preincubation. We conclude that TIR1 protein is a rate‐limiting factor for target protein depletion in yeast, and we provide new tools that allow tightly controlled, tuneable, and efficient depletion of essential proteins whereas minimising secondary effects.

## 1 INTRODUCTION

A common approach to study the function of an essential gene *in vivo* is to investigate the consequence of conditionally repressing its expression. Ideally, this approach should result in a fast and specific repression to minimise secondary and off‐target effects. The auxin‐inducible degron (AID; Nishimura, Fukagawa, Takisawa, Kakimoto, & Kanemaki, 2009) is a technique that can fulfil this goal as it allows fast depletion of the target protein by incubating the cell culture with a small molecule (auxin) that does not perturb cell metabolism. The physiological role of auxin is to regulate growth and development in plants, through a pathway that leads to the proteasome‐mediated degradation of the Aux/IAA family of transcriptional regulators (reviewed in Teale, Paponov, & Palme, 2006). This pathway can be artificially transferred to a nonplant organism by expressing the TIR1 gene, which encodes a plant auxin‐binding receptor that interacts with the conserved E3 ubiquitin ligase SCF complex. The target gene is fused with a transcriptional repressor Aux/IAA protein that functions as the degron tag (domain to induce degradation). When TIR1 protein is bound to auxin, it interacts with the degron, allowing the E3 ubiquitin ligase SCF complex to polyubiquitinate the target protein, thereby directing its degradation by the proteasome.

In the first version of the AID system for yeast, which was developed by Nishimura et al. (2009), expression of Oryza sativa *TIR1* (OsTIR1) was driven by a galactose‐inducible *(GAL1–10*) promoter or the constitutive *ADH1* promoter. The galactose‐inducible expression system can result in metabolic perturbations caused by the shift from glucose to galactose as carbon source (Bergkessel, Whitworth, & Guthrie, 2011; Kresnowati et al., 2006; Ronen & Botstein, 2006). Here, we tested the constitutive expression of Os*TIR1* (from here on referred to as TIR1)*,* encoded by a codon‐optimized sequence from the Kanemaki lab, under the control of a strong (P*adh1–701*) or weak (P*adh1–*409) promoter (Santangelo & Tornow, 1990) and show that constitutive expression of this TIR1 is problematic. High expression can result in uncontrolled target protein depletion (even in the absence of auxin). Conversely, a weak promoter can lead to slow depletion when TIR1 is expressed at low levels. This suggests that it is important to express TIR1 in a tightly controlled manner in order to achieve optimal results.

McIsaac et al. (2013) developed a β‐estradiol‐inducible budding yeast expression system that makes use of an artificial transcription factor, ZnEV, made by fusing a human estrogen receptor (ER), with the viral transcription activator VP16 and three or four (where *n* = 3 or 4) zinc finger DNA‐binding domains that recognise a specific promoter, ZnEVpr. In the absence of estrogen (β‐estradiol), the Hsp90 chaperone complex inhibits ZnEV by interacting with its ER domain. Binding of β‐estradiol to ER releases it from the Hsp90 complex, allowing ZnEV to transcriptionally activate ZnEVpr, through direct interaction between the zinc‐finger array of ZnEV and its target DNA sequence within ZnEVpr (reviewed in Pratt & Toft, 1997). McIsaac et al. (2013) report ∼100‐fold increase in the level of a green‐fluorescent reporter protein that is produced under control of ZnEVpr only 30 min after addition of β‐estradiol. More importantly, they show that the system is specific, as incubating cells with β‐estradiol does not affect the global transcription profile of the yeast cells.

Here, we present a new version of the AID system for budding yeast that we call “β‐est AID,” in which expression of TIR1 is tightly regulated by the β‐estradiol system. To test this system, we measured initial levels and depletion rate of several target proteins in a strain of Saccharomyces cerevisiae harbouring the β‐est AID and compared them with those of strains that constitutively express TIR1. We then compared depletion rates of several nuclear target proteins following preincubation with β‐estradiol for different times. The results show that depletion rate directly correlates with length of preincubation with β‐estradiol and, therefore, with TIR1 levels. We demonstrate that even a highly abundant target protein can be quickly depleted by extending the preincubation with β‐estradiol. Finally, we show that the β‐est AID system works when the components are genomically encoded, but for ease of use, we constructed a plasmid that allows all the elements of the β‐est AID system to be introduced into budding yeast through a one‐step transformation.

## 2 MATERIALS AND METHODS

### 2.1 Plasmids

pMK200 (NBRP ID: BYP7569; Masato Kanemaki) was sourced from the Yeast Genetic Resource Centre (YGRC; Osaka University). pHyg‐AID* plasmids were a gift from the Ulrich lab (Morawska & Ulrich, 2013). pBRT1 was constructed with the following DNA parts: natMX6 backbone plasmid, 409‐base pair truncation of the *ADH1* promoter (P*adh1–409*), yeast codon‐optimized *TIR1* from pMK200, and *HIS3*‐flanking sequences for genomic insertion. To generate plasmids pURA3‐AID*‐6FLAG, pURA3‐AID*‐9myc, and pURA3‐AID*‐6HA, the HPH marker in pHyg‐AID* plasmids was replaced with the *Kluyveromyces lactis URA3* gene flanked by a 142 nt repeat sequence from its 5’UTR, which allows selective pop‐out of the *URA3* sequence by growth in 5‐fluoroorotic acid. pZTRL was constructed by Gibson Assembly with the following DNA parts: pRS415 (CEN/ARS LEU2), Z_4_EVpr from pMN10 (McIsaac et al., 2013), yeast codon‐optimized *TIR1* from pMK200, and *ACT1*p‐Z4EV from yeast strain YMN3 (McIsaac et al., 2013). pZTRK was made by replacing the *LEU2* selection marker in pZTRL with KanMX. pMN10 was kindly provided by R. Scott McIsaac. Snapgene‐generated plasmid maps (Supplemental Figure S1) and sequences of pBRT1, pURA3‐AID*‐6FLAG, pURA3‐AID*‐9myc, pURA3‐AID*‐6HA, pZTRK, and pZTRL can be found in the Supporting Information. These plasmids will be deposited to YGRC (http://yeast.nig.ac.jp/yeast/). The plasmid map displayed in Figure 4a was generated using Plasmid Drawing Program Plasmidomics 0.2 (Dr. Robert Winkler).

### 2.2 Yeast strains and growth conditions

Yeast strains are listed in Supplemental Table S1 (These strains will be deposited to YGRC (http://yeast.nig.ac.jp/yeast/). PADH1–701‐TIR1 was made by inserting *Stu*I‐linearized pMK200 (containing P*adh1–701*‐Os*TIR1 URA3* expression cassette) into the *ura3–1* locus of W303, whereas PADH1–409‐TIR1 was made by inserting the 4398 bp product of *PmI*1‐linearized pBRT1 (which contains natMX P*adh1–409*‐Os*TIR1* expression cassette) into the *his3–11,15* locus of W303. PZ4EV‐NTIR1 was created by inserting pKanMX‐Z_4_EVpr (from pMN10) directly upstream of the start codon of *APE2*, as described by McIsaac et al. (2013), followed by *URA3* pop‐in/pop‐out substitution of *APE2* protein‐coding sequence for NLS‐Os*TIR1*‐V5. PZ4EV‐TIR1 differs from PZ4EV‐NTIR1 only in that it lacks the SV40 NLS in the N‐terminus of *OsTIR*‐V5. *PRP22*, *PRP2*, *DCP1, YHC1*, and *RRP44* were AID*‐tagged by transforming PADH1–701‐TIR1, PADH1–409‐TIR1, PZ4EV‐NTIR1, PZ4EV‐TIR1, or pZTRL‐bearing W303, with pHyg‐AID*‐ or pURA3‐AID*‐ cassettes, following a PCR‐based method (Longtine et al., 1998). YMN3 was kindly provided by R. Scott McIsaac. Yeast were grown at 30°C on Yeast Peptone Dextrose supplemented with adenine (YPDA) or yeast minimal media (YMM) supplemented with Kaiser drop outs (Formedium). When required, β‐estradiol (Sigma‐Aldrich) was added at a final concentration of 10 uM and indole‐3‐acetic acid (auxin; Acros Organics) at a final concentration of 750 uM. Samples of yeast cultures were fixed in methanol, chilled in dry ice at a ratio of 3:2 (culture:methanol by volume).

### 2.3 Western blots

Protein extracts were prepared following a NaOH/TCA precipitation method (Volland, Urban‐Grimal, Géraud, & Haguenauer‐Tsapis, 1994). Protein concentrations were measured by the Bradford method and equal amounts of protein were loaded for each sample into SDS‐PAGE. Proteins were transferred to Immobilon‐FL PVDF (Millipore, Cat. No. IPFL00010) and probed with rat anti‐FLAG (Agilent, Cat. No. 200474), mouse anti‐HA (Roche, Cat. No. 11583816001), mouse anti‐PGK1 (Abcam, Cat. No. Ab113687) and/or mouse anti‐V5 (Invitrogen, Cat. No. MA5–15253); and LI‐COR secondary antibodies (Cat. No. 925–32219, 925–68020 and/or 925–32210). Rabbit anti‐OsTIR1 antibody was provided by Masato Kanemaki. Blots were developed with the LI‐COR Odyssey system and images analysed using the Odyssey Image Studio software to quantify the band signals. Normalisation was by amount of protein loaded. Pgk1 bands were also quantified for comparison.

### 2.4 Reverse transcription‐quantitative polymerase chain reaction

RT‐qPCR was performed as described in (Alexander et al., 2010). Primers for RT‐qPCR were TTGCTGCAAGATTCCCAAACG (TIR1 167F), CCCCAATCAGGTGGAACCAA (TIR1 254R), TAAGCTGGCATGTGCTGCATTC (ALG9_F), and TTTGCATGATTCGGTTGATTGG (ALG9_R). Transcript copy numbers per cell were estimated by comparing the relative abundance of TIR1 amplicon to that of internal control *ALG9* (1.28 copies/cell; Miura et al., 2008; Teste, Duquenne, François, & Parrou, 2009).

## 3 RESULTS

### 3.1 High‐level expression of TIR1 causes auxin‐independent depletion of target protein

**Figure 1:**
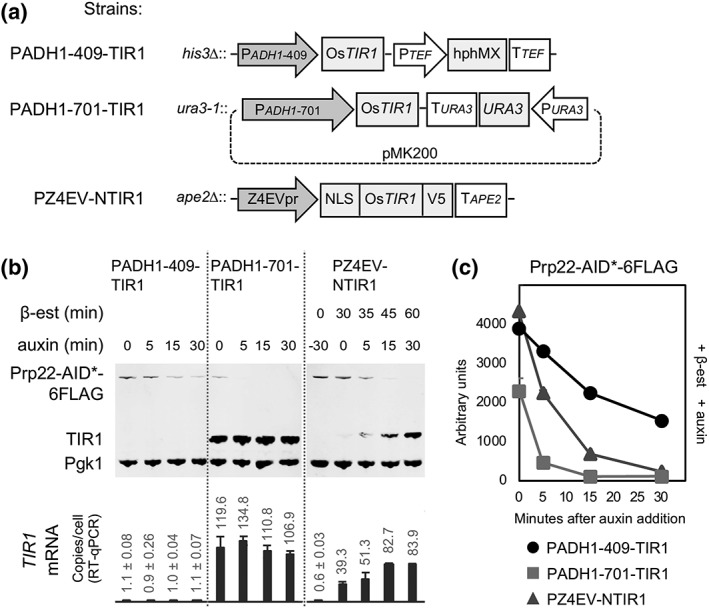
High‐level expression of TIR1 causes auxin‐independent depletion of target protein. (a) genomically integrated *TIR1*‐expression cassettes of strains, PADH1–409‐TIR1, PADH1–701‐TIR1, and PZ4EV‐NTIR1 used in this study. Z4EVpr promoter is induced by β‐estradiol (β‐est) whereas P*adh1* promoters are constantly active. (b) Upper: Western blot of Prp22‐AID*‐6FLAG, TIR1, and Pgk1 (as visual loading control; equal amounts of total protein were loaded in each lane) at different times (min; minutes) of β‐estradiol and/or auxin incubation; lower: estimated copies/cell of *TIR1* transcripts from the corresponding culture samples. (c) quantification of western blot shown in panel B

First, we compared strains PADH1–701‐TIR1 and PADH1–409‐TIR1 that constitutively express TIR1 to high or low levels, respectively (Figure 1a,b). To this end, we C‐terminally tagged splicing factor Prp22 with a fusion between a truncated auxin‐dependent degron, named AID* (Morawska & Ulrich, 2013) and six tandem repeats of the FLAG epitope, in strains PADH1–701‐TIR1 and PADH1–409‐TIR1. To measure the rate of target protein depletion, we added auxin (indole‐3‐acetic acid) to the cultures (time 0), and samples were taken after 5, 15, and 30 min and snap frozen.

Quantification of proteins in the samples (Figure 1b,c) shows that at time 0 (no auxin), the levels of Prp22 were 47% lower in PADH1–701‐TIR1 than in PADH1–409‐TIR1. As PADH1–701‐TIR1 constitutively expresses TIR1 to a higher level, this may indicate that too much TIR1 can cause uncontrolled depletion of the target protein. Also, the Prp22 depletion rate was higher in PADH1–701‐TIR1 than in PADH1–409‐TIR1. By 30 min after auxin addition, Prp22 levels had dropped to below 6% of the initial values in PADH1–701‐TIR1, compared with 35% remaining in PADH1–409‐TIR1, suggesting that high levels of TIR1 promote faster depletion.

We, therefore, created an inducible‐TIR1 strain by placing the *OsTIR1* gene under control of the Z4EV promoter (Z4EVpr). This was done by replacing the nonessential *APE2* gene in the Z4EV‐expressing strain YMN3 (McIsaac et al., 2013) with a Z4EVpr‐Os*TIR1*‐V5 cassette (Figure 1a). To promote localisation of TIR1 protein to the nucleus, where our protein targets are located, we fused an SV40 nuclear localisation signal (NLS) to the *TIR1*‐coding sequence. The resulting strain, PZ4EV‐NTIR1, produces TIR1‐V5 protein rapidly after addition of β‐estradiol to the culture medium (Figure 1b), and auxin was added after preinduction of TIR1 for 30 min. Under these conditions, Prp22 was depleted rapidly after auxin addition, without detectable auxin‐independent depletion (Figure 1b,c).

**Figure 2:**
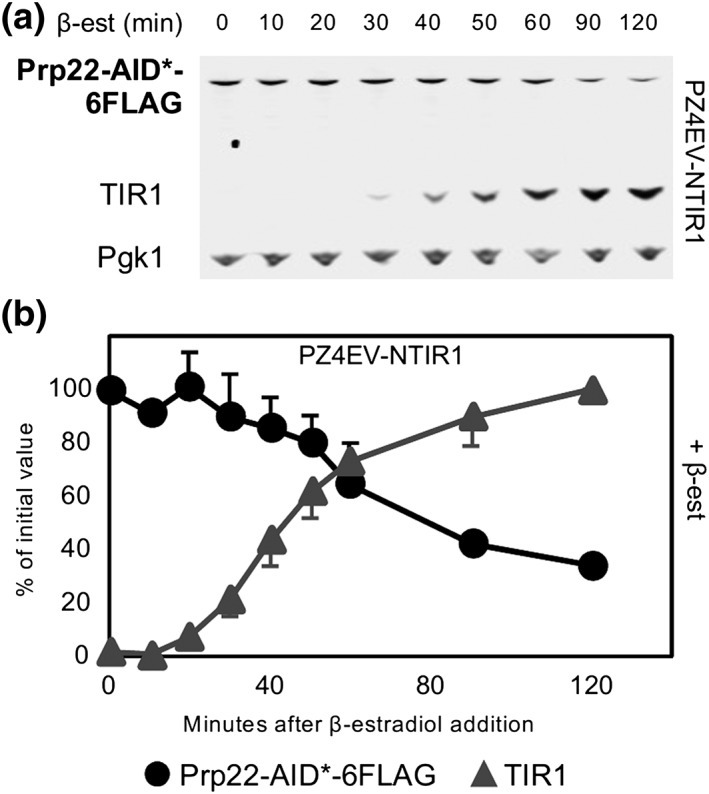
Auxin‐independent depletion can be controlled by inducible expression of TIR1. (a) Western blot of AID‐target Prp22‐AID*‐6FLAG and β‐estradiol‐inducible TIR1 (strain PZ4EV‐NTIR1), after addition of β‐estradiol but without auxin. (b) Quantification of western blot shown in panel A. Error bars represent standard deviation (sd) of two biological replicates. For each experiment, only one representative blot is shown. Error bars represent standard deviation (sd) of two biological replicates. For each experiment, only one representative blot is shown

Next, to test the hypothesis that levels of TIR1 inversely correlate with levels of the target protein in the absence of auxin, a culture of PZ4EV‐NTIR1 with AID*‐6FLAG‐tagged *PRP22* was incubated with β‐estradiol but without auxin, and the levels of Prp22 were measured over time. Consistent with our previous observation, at 50 min of β‐estradiol incubation, the level of Prp22 had dropped significantly and reached 35% of the initial value at 2 hr of incubation, by which time the TIR1‐V5 protein was well induced (Figure 2). Auxin‐independent depletion of Yhc1 and Rrp44 is shown in Figure S2 and was less pronounced with the more abundant Rrp44. We conclude that high levels of TIR1 can cause auxin‐independent depletion of the target protein in budding yeast.

### 3.2 Depletion rate can be tuned by modulating the duration of β‐estradiol preincubation

Next, we investigated how the rate of auxin‐induced depletion is influenced by the length of β‐estradiol preincubation. On the basis of the previous results, we anticipated that not only longer preincubation times would lead to faster depletion but also more auxin‐independent depletion and that there may be an optimal preincubation time, which is likely to be target specific. To test this, we used as targets for depletion, Prp22 (232 copies/cell), Prp2, another essential splicing factor with similar abundance (211 copies/cell), and the more highly expressed decapping enzyme, Dcp1 (4,189 copies/cell; Kulak, Pichler, Paron, Nagaraj, & Mann, 2014). We performed a time‐course depletion analysis in which cultures of these AID*‐tagged strains were preincubated with β‐estradiol for different times (20, 30, 40, or 60 min) prior to auxin addition. Samples were then taken for protein analysis at 5‐min intervals. As the levels of TIR1 increase with time of β‐estradiol incubation, this allowed us to measure the relationship between TIR1 abundance and auxin‐dependant depletion rate of different target proteins.

**Figure 3:**
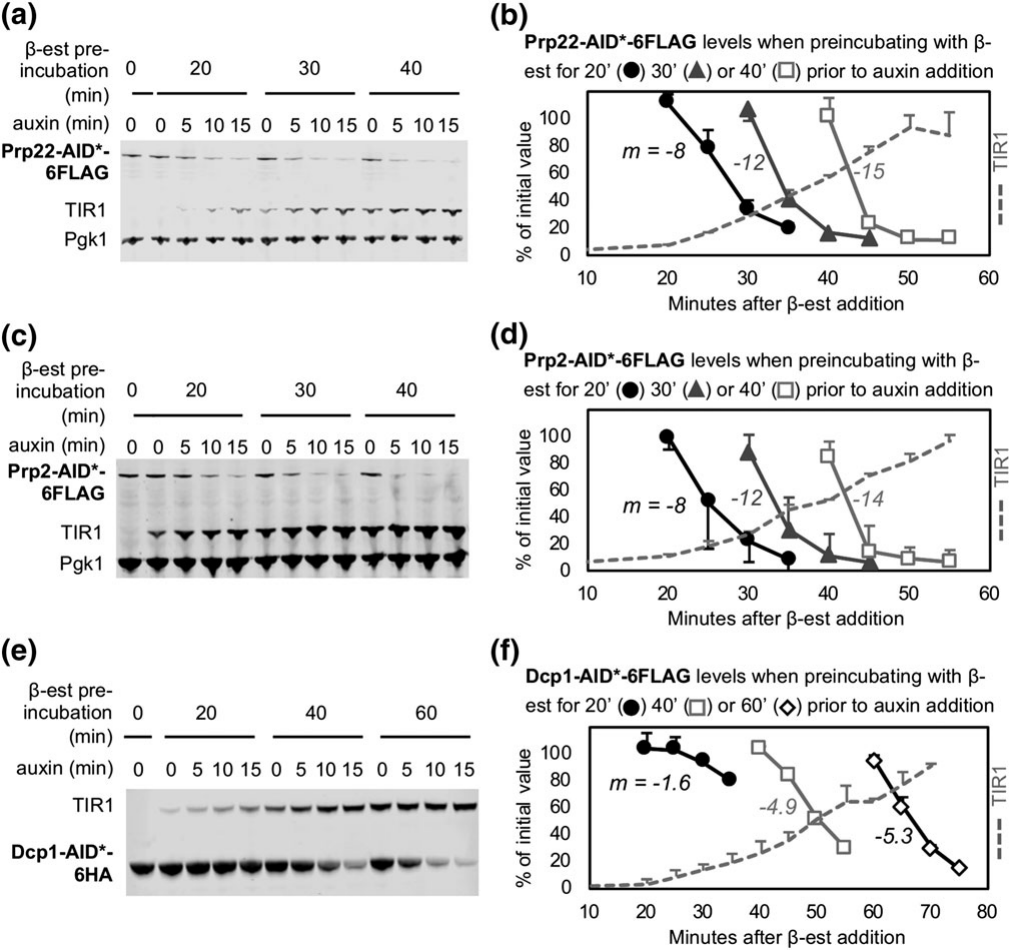
Depletion rate can be tuned by modulating the duration of β‐estradiol pre‐incubation. Western blot of AID‐targets (a–b) Prp22‐AID*‐6FLAG, (c–d) Prp2‐AID*‐6FLAG, and (e–f) Dcp1‐AID*‐6HA, from cultures preincubated with β‐estradiol (β‐est) for 20, 30, 40, or 60 min prior to auxin addition. Equal amounts of total protein were loaded in each lane, and Pgk1 is included as a visual loading control, except for panel E, where Pgk1 and Dcp1 comigrate. Quantifications of protein bands in panels A, C, and E are shown in panels B, D, and F. As a measure of depletion rate, the slope (m) was calculated for the linear section (from 100 to 30% of initial values) of each curve. Error bars represent sd of two biological replicates. For each experiment, only one representative blot is shown

The protein quantification analysis (Figure 3), shows that different target proteins were depleted at different rates. A 20‐min preincubation with β‐estradiol was sufficient to reduce Prp22 and Prp2 to low levels (≤ 20%) within 15 min of auxin addition, but longer preincubations with β‐estradiol resulted in auxin‐independent degradation. In contrast, the more abundant Dcp1 required 60‐min of β‐estradiol preincubation to achieve a similarly rapid and efficient depletion because more Dcp1 protein has to be degraded to achieve efficient depletion in terms of percentage of the starting amount, and this evidently requires more TIR1 protein. Notably, with all three target proteins, there was a direct correlation between the duration of β‐estradiol preincubation and the depletion rate so that the duration of β‐estradiol treatment should be optimised for each target protein (see also Figure S2).

### 3.3 Plasmid‐encoded β‐est AID and the effect of TIR1 protein localization on efficiency of targeted depletion

**Figure 4:**
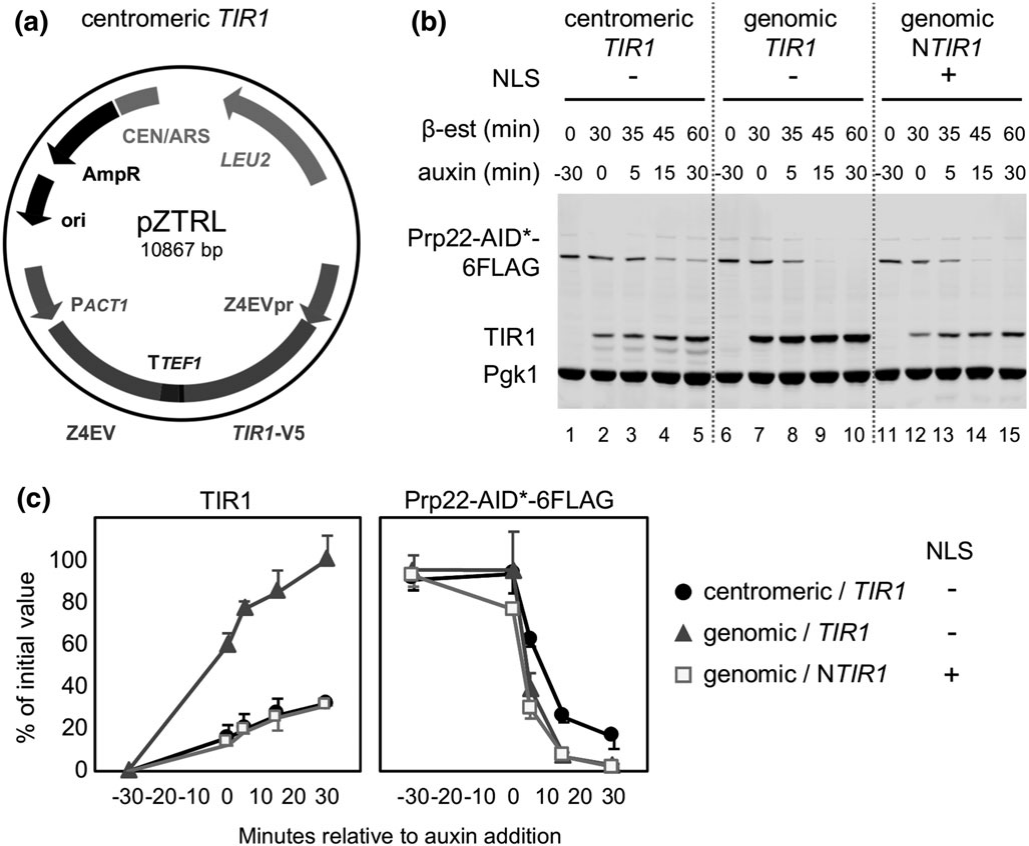
Plasmid‐encoded β‐est AID and the effect of TIR1 protein localization on targeted depletion efficiency. (a) Plasmid map of pZTRL. (b) Western blot of TIR1 and AID‐target Prp22‐AID*‐6FLAG in three different strains preincubated with β‐estradiol for 30 min (min) prior to auxin addition. Equal amounts of total protein were loaded in each lane, and Pgk1 is included as a visual loading control. In the first strain (lanes 1–5), both Z4EVpr‐Os*TIR1*‐V5 and P*act1*‐Z4EV expression cassettes are located in pZTRL centromeric plasmid; whereas in strains PZ4EV‐TIR1 (lanes 6–10) and PZ4EV‐NTIR1 (lanes 11–15), they are genomically encoded. In PZ4EV‐NTIR1, an SV40 nuclear localization signal was included in the N‐terminal of *TIR1*. (c) Quantification of western blot signal shown in panel B. Error bars represent sd of two biological replicates. Only one representative blot is shown

To facilitate insertion of the β‐est AID components into budding yeast, we constructed a centromeric plasmid, pZTRL, which contains P*act1*‐Z4EV and Z4EVp‐Os*TIR1* (without NLS) expression cassettes (Figure 4a). We then tested pZTRL and, at the same time, investigated the effect of fusing an NLS to TIR1 on the efficiency of depleting a nuclear protein. To this end, we measured both TIR1 and Prp22 target protein levels in the pZTRL‐bearing strain, and in two strains that contain genomically integrated *TIR1*, with (PZ4EV‐NTIR1) or without (PZ4EV‐TIR1) NLS on the N‐terminal of TIR1 (Figure 4b,c).

We observed that the β‐estradiol‐induced level of TIR1 protein was about threefold higher in the genomic *TIR1* (PZ4EV‐TIR1) strain compared with genomic NLS‐*TIR1* (PZ4EV‐NTIR1) or plasmid‐encoded *TIR1* strains, indicating that *TIR1* is better expressed when it is genomically‐integrated and lacks an NLS. Interestingly, Prp22, which is a nuclear localized protein, was quickly depleted irrespective of the presence or absence of the NLS in the N‐terminus of TIR1 protein. This suggests that, for depletion of nuclear proteins, an SV40 NLS need not be added to TIR1. Notably, targeted depletion was slower in the plasmid‐encoded TIR1 strain compared with the other two strains, reaching 15% compared with 2% of Prp22 initial values 30 min after auxin addition, probably due to a lower expression of TIR1 in this strain.

## 4 DISCUSSION

Our observation that in budding yeast high levels of TIR1 protein cause auxin‐independent depletion agrees with reports that over‐expression of TIR1 in Arabidopsis thaliana leads to an auxin‐response phenotype (Gray et al., 1999) and depletion of TIR1 substrates (Aux/IAA proteins; Dos Santos Maraschin, Memelink, & Offringa, 2009), even without exogenous auxin. More recently, Natsume, Kiyomitsu, Saga, and Kanemaki (2016) constructed a tetracycline‐inducible TIR1 for human cells and showed that incubation with the tetracycline analogue doxycycline slows growth of a DHC1‐AID tagged cell culture, implying that in human cells, over‐expression of TIR1 may cause leaky depletion of the target (Natsume et al., 2016). In our work, we were repeatedly unsuccessful with 10 out of 20 essential genes that we tried to AID‐tag in PADH1–701‐TIR1 (high‐level TIR1 expression), whereas eight of these were successfully tagged in either PADH1–409‐TIR1 (low‐TIR1 expression) or in pZTRL. Indeed, all 22 essential genes for which AID tagging was attempted in PADH1–409‐TIR1 (low‐TIR1 expression), and all nine attempts to AID‐tag essential genes in pZTRL were successful (data not shown), with success defined as failure to grow in the presence of auxin. This, together with our measurements of Prp22 and TIR1 levels in these strains, strongly suggests that high‐level expression of TIR1 causes auxin‐independent degradation that may result in target proteins falling below the level required for viability. The auxin independent activity of SCF‐TIR1 E3 ubiquitin ligase complex that we and others have observed could be caused by low‐affinity interaction of TIR1 with its target in the absence of auxin (Dharmasiri, Dharmasiri, & Estelle, 2005; Kepinski & Leyser, 2005; Tan et al., 2007); by traces of auxin in the media, as previously speculated (Natsume & Kanemaki, 2017); or by low levels of endogenous auxin in plants and yeast (Rao, Hunter, Kashpur, & Normanly, 2010).

Notably, the plasmid‐encoded, β‐est AID (pZTRL) depleted the target protein more slowly than genomically integrated TIR1 (Figure 4), likely because the plasmid‐encoded TIR1 protein was produced at a lower level. This could be due to transcriptional interference between P*act1*‐Z4EV and Z4EVpr‐Os*TIR1* that are convergently transcribed in pZTRL, for example, if their shared transcriptional terminator T*TEF1* does not act bidirectionally—even though it was proposed that most transcriptional terminators in S. cerevisiae function bidirectionally (Uwimana, Collin, Jeronimo, Haibe‐Kains, & Robert, 2017). Thus, the plasmid‐based β‐est AID may require longer β‐estradiol preincubation times and may not be ideal for protein targets that are more abundant. In addition to pZTRL, we created pZTRK by replacing *LEU2* in pZTRL with KanMX. pZTRK can be propagated in rich media, which is useful given that high concentrations of auxin can impair growth in minimal media (T.S. Turowski, S. Bresson and D. Tollervey, personal communication).

We targeted only nuclear proteins for depletion, which is why we tested the effect of an NLS sequence at the start of the TIR1 protein. However, as previously reported for budding yeast (Tanaka, Miyazawa‐Onami, Lida, & Araki, 2015), the presence of the NLS had little effect on depletion rate of a nuclear protein (Prp22). We speculate that TIR1 protein when N‐terminally fused to an NLS may have reduced stability, but that this may result in similar amounts of nuclear localised TIR1 protein in the presence or absence of an NLS, such that the target protein depletion rates are similar. Nishimura et al. (2009) showed that the AID system allows the rapid and efficient depletion of proteins present either in the nucleus or the cytoplasm. Therefore, we anticipate that our non‐NLS TIR1 would also support efficient depletion of cytoplasmic targets although we have not tested this.

In summary, we present evidence that the level of expression of TIR1 has a profound influence on how rapidly and completely the AID system depletes its target protein. In order to enhance this system for the molecular biology community, we developed a suite of strains and plasmids in S. cerevisiae. The strain PADH1–409‐TIR1 allows a slower, more linear depletion of the target protein (Figure 1c), which is useful where full depletion is undesirable, and may facilitate kinetic studies of the effects of protein depletion. The β‐estradiol‐induced TIR1 strains can have their preincubation time optimised to produce an extremely rapid and effective degradation of the target protein whereas minimising auxin‐independent degradation. Although this tunable β‐estradiol expression system has been developed specifically for use in budding yeast, the data and principles we present are likely to apply to other organisms and to be useful for the wider scientific community interested in making the most out of the powerful AID technique.

## Supporting information

Table S1. Supporting Information

Figure S1. Plasmid maps

Figure S2. Time courses of Yhc1 and Rrp44 depletion with and without addition of auxin. Yhc1 and Rrp44 were AID*‐tagged in strain PZ4EV‐NTIR1 as described in Materials and Methods. β‐estradiol was added at time 0 (T0) either without auxin (left panels) or with auxin addition at a previously determined optimal time (30 min for Yhc1 and 40 min for Rrp44; right panels). Data represent western blot quantifications relative to initial values.
