# Supplementary material for: A fast and tuneable auxin‐inducible degron for depletion of target proteins in budding yeast
Source: Yeast. 2018 Nov 12;36(1):75–81. doi: 10.1002/yea.3362 (PMC6587778; doi:10.1002/yea.3362)
Supplement: Supplementary file 1 — Table S1. Supporting Information [file YEA-36-75-s001.docx]

Mendoza-Ochoa et al.

**Table S1**

| **Strain** | **Genotype** | **Parental strain** | **Source** |
| --- | --- | --- | --- |
| W303 | MATα *ade2-1 ura3-1 his3-11,15 trp1-1 leu2-3,112 can1-100* | - | - |
| PADH1-701-TIR1 | MATα *ade2-1 his3-11,15 trp1-1 leu2-3,112 can1-100 ura3-1*::P*ADH1*-701-Os*TIR1-URA3* | W303 | This study |
| PADH1-409-TIR1 | MATα *ade2-1 ura3-1 trp1-1 leu2-3,112 can1-100 his3-11,15::*P*ADH1*-409-Os*TIR1*-*NatMX* | W303 | This study |
| YMN3 | MATα *ura3*∆ *leu2*∆0::P*ACT1*-Z4EV-NatMX | - | S. McIsaac |
| PZ4EV-NTIR1 | MATα *ura3∆ leu2*∆0::P*ACT1*-Z4EV-NatMX *ape2*::Z4EVpr-NLS-Os*TIR1*-V5 | YMN3 | This study |
| PZ4EV-TIR1 | MATα *ura3∆ leu2*∆0::P*ACT1*-Z4EV-NatMX *ape2*::Z4EVpr-Os*TIR1*-V5 | YMN3 | This study |
| Prp22-AID*-6FLAG_PADH1-701-TIR1 | MATα *ade2-1 his3-11,15 trp1-1 leu2-3,112 can1-100 ura3-1*::P*ADH1*-701-Os*TIR1-URA3 PRP22::PRP22*-AID*-6FLAG-HygMX | PADH1-701-TIR1 | This study |
| Prp22-AID*-6FLAG_PZ4EV-NTIR1 | MATα *ura3∆ leu2*∆0::P*ACT1*-Z4EV-NatMX *ape2*::Z4EVpr-NLS-Os*TIR1*-V5 *PRP22*::*PRP22*-AID*-6FLAG-HygMX | PZ4EV-NTIR1 | This study |
| Prp22-AID*-6FLAG_PADH1-409-TIR1 | MATα *ade2-1 ura3-1 trp1-1 leu2-3,112 can1-100 his3-11,15::*P*ADH1*-409-Os*TIR1-NatMX PRP22::PRP22*-AID*-6FLAG-HygMX | PADH1-409-TIR1 | This study |
| Prp2-AID*-6FLAG_PZ4EV-NTIR1 | MATα *ura3∆ leu2*∆0::P*ACT1*-Z4EV-NatMX *ape2*::Z4EVpr-NLS-Os*TIR1*-V5 *PRP2*::*PRP2*-AID*-6FLAG | PZ4EV-NTIR1 | This study |
| Dcp1-AID*-6HA_PZ4EV-NTIR1 | MATα *ura3∆ leu2*∆0::P*ACT1*-Z4EV-NatMX *ape2*::Z4EVpr-NLS-Os*TIR1*-V5 *DCP1*::*DCP1*-AID*-6HA | PZ4EV-NTIR1 | This study |
| Prp22-AID*-6FLAG_PZ4EV-TIR1_pRS415 | MATα *ura3∆ leu2*∆0::P*ACT1*-Z4EV-NatMX *ape2*::Z4EVpr-Os*TIR1*-V5 *PRP22*::*PRP22*-AID*-6FLAG-HygMX [pRS415] | PZ4EV-TIR1 | This study |
| Prp22-AID*-6FLAG_PZ4EV-NTIR1_pRS415 | MATα *ura3∆ leu2*∆0::P*ACT1*-Z4EV-NatMX *ape2*::Z4EVpr-NLS-Os*TIR1*-V5 *PRP22*::*PRP22*-AID*-6FLAG-HygMX [pRS415] | YMN3 | This study |
| Prp22-AID*-6FLAG_pZTRL | MATα *ade2-1 ura3-1 his3-11,15 trp1-1 leu2-3,112 can1-100 PRP22::PRP22-AID*-6FLAG-HygMX* [pZTRL] | W303 | This study |
| N.B. Prp22-AID strains with integrated TIR1 or NTIR1 carry plasmid pRS415 (CEN, *LEU2*) to permit growth without leucine, allowing direct comparison with the strain carrying pZTRL  N.B.B. These strains and plasmids will be deposited to YGRC (<http://yeast.nig.ac.jp/yeast/>). | | | |
