# Supplementary figures and images for: A fast and tuneable auxin‐inducible degron for depletion of target proteins in budding yeast
Source: Yeast. 2018 Nov 12;36(1):75–81. doi: 10.1002/yea.3362 (PMC6587778; doi:10.1002/yea.3362)

Figure S1: Plasmid maps

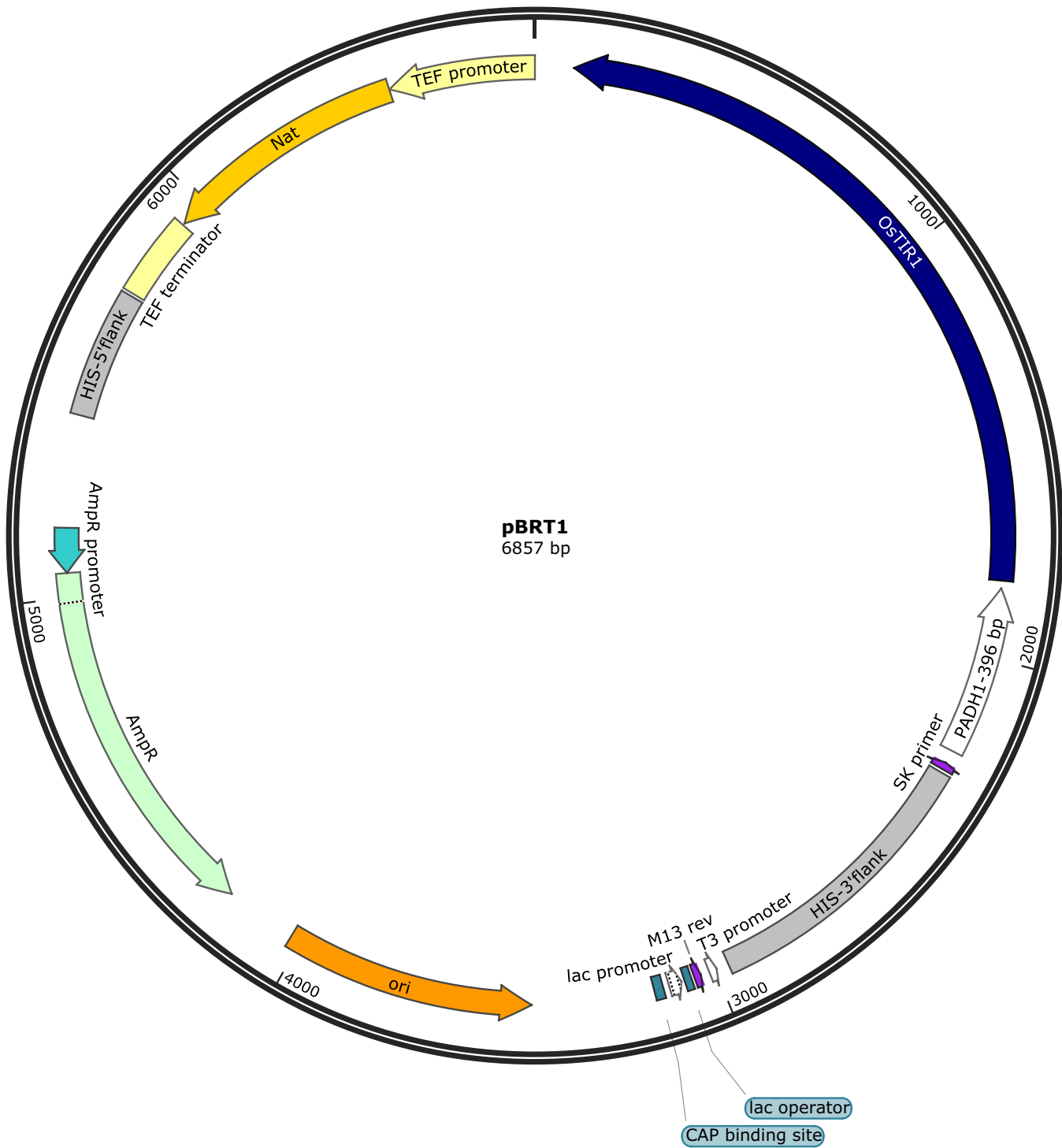

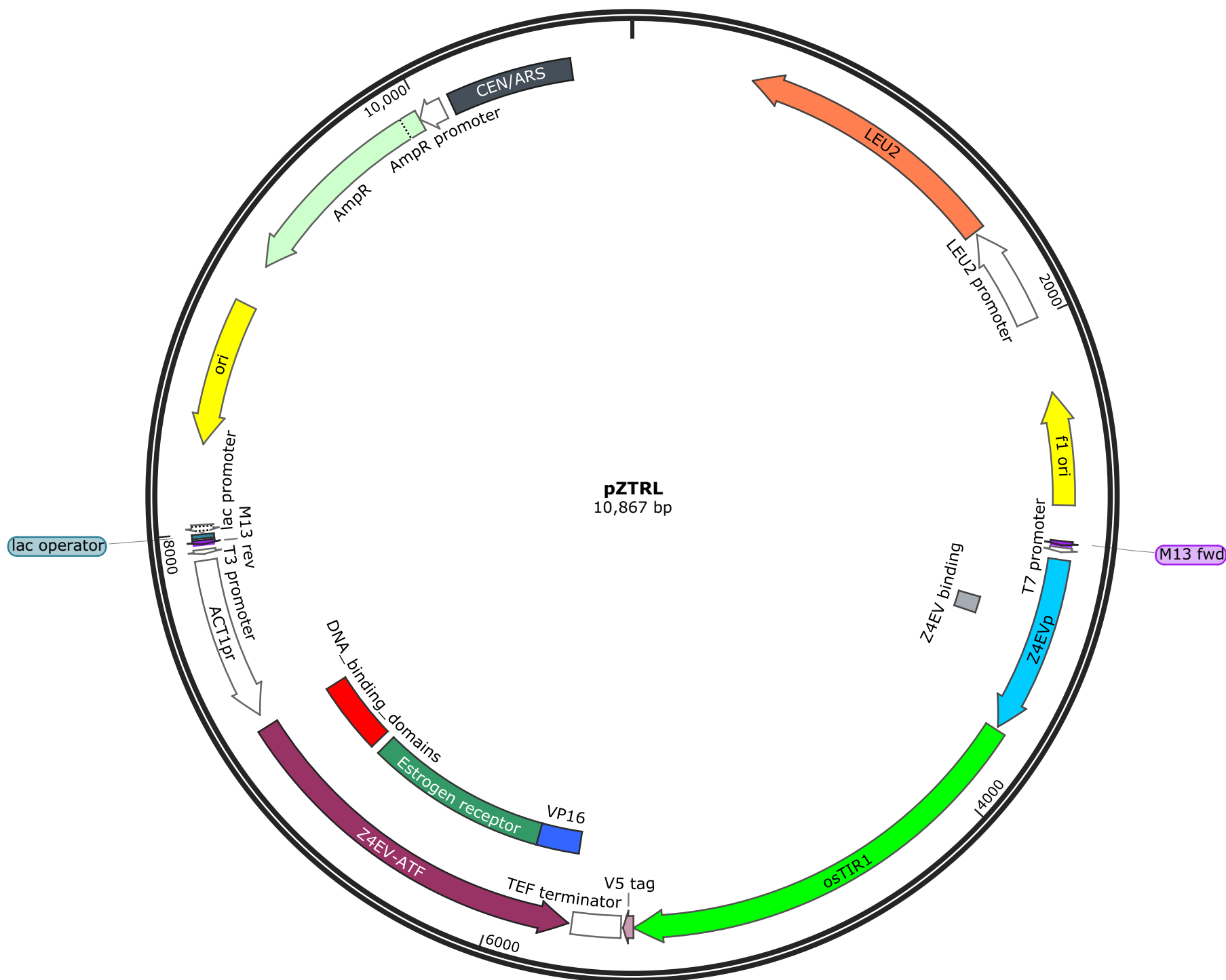

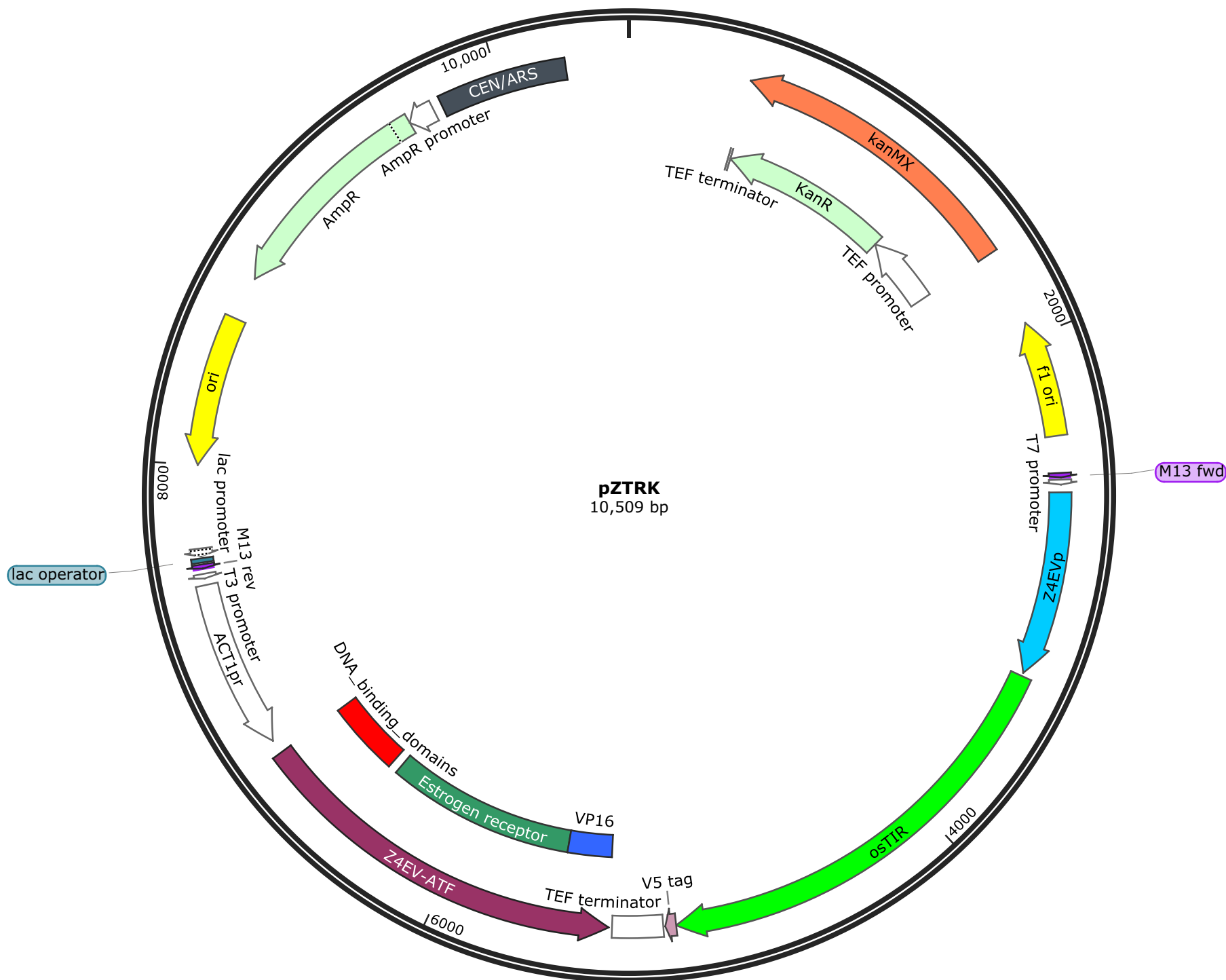

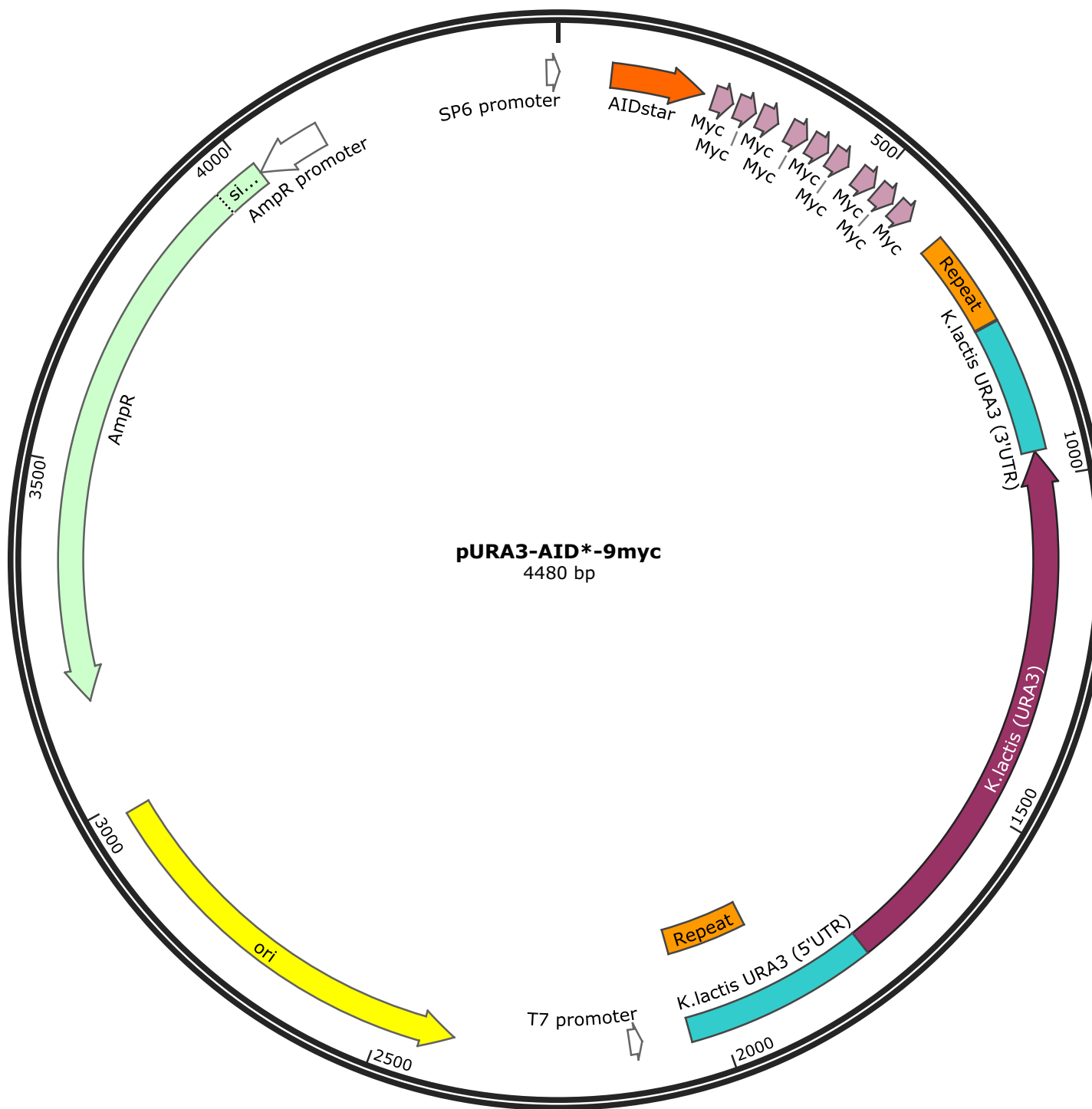

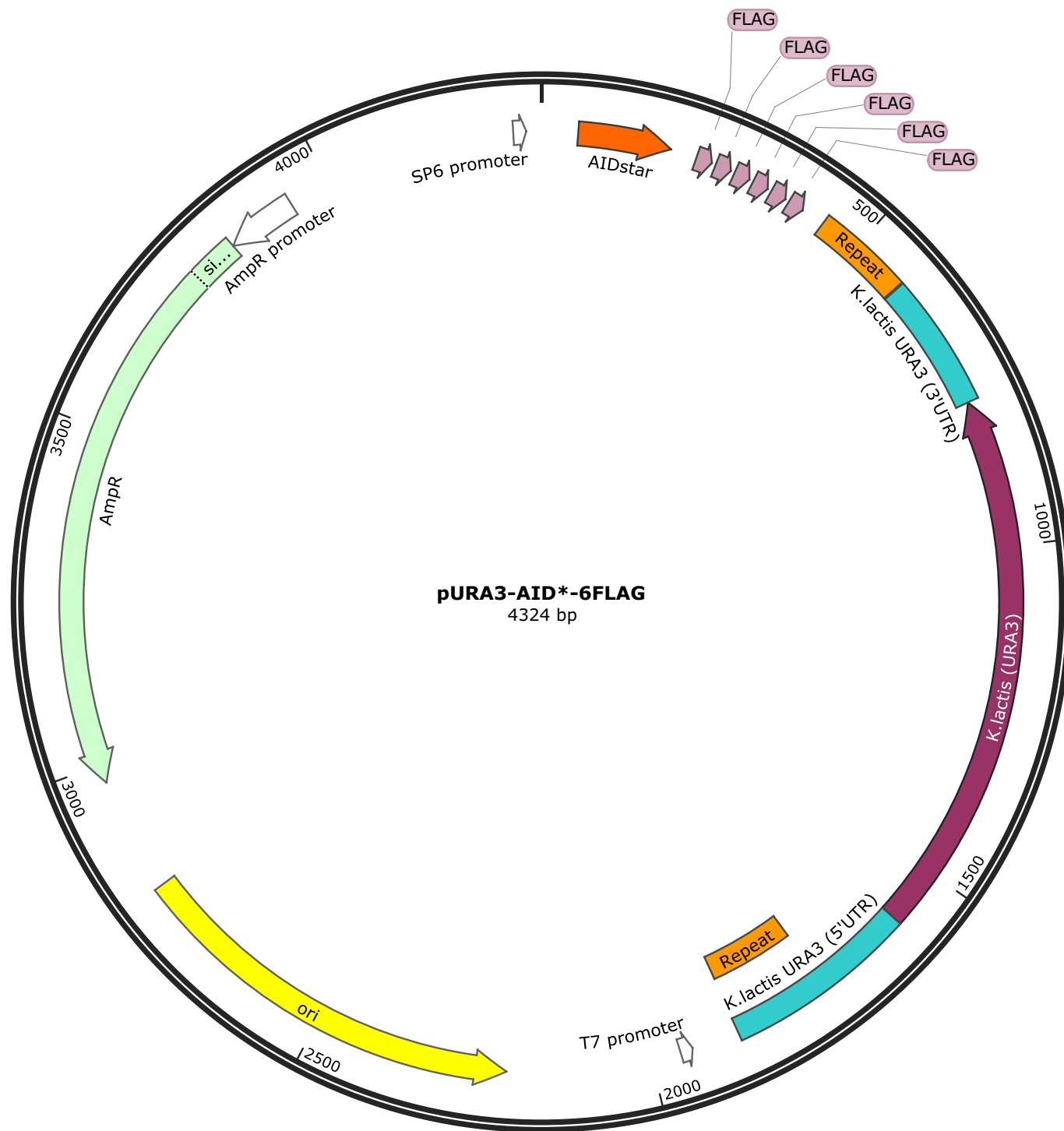

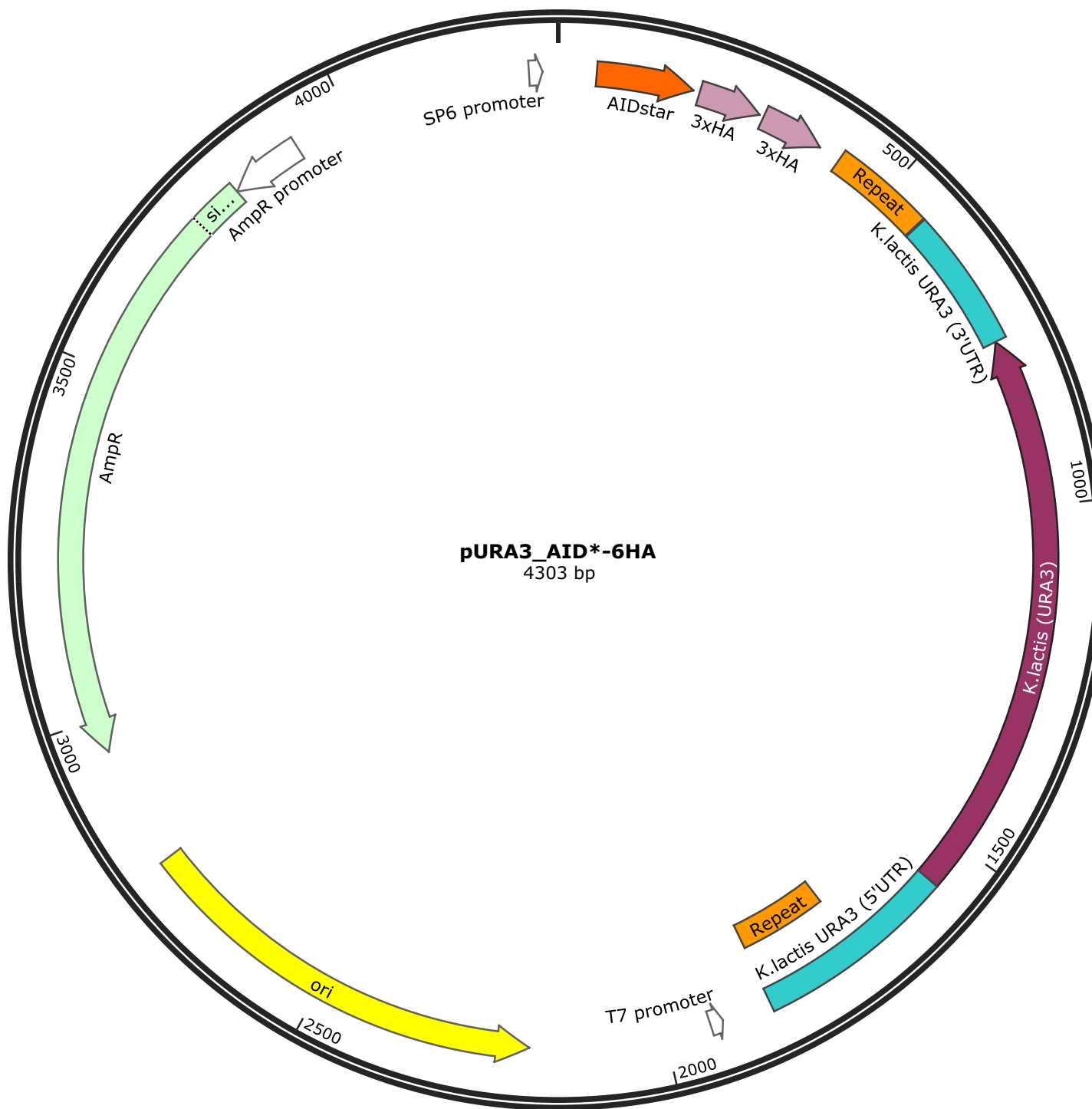

Supplement: Supplementary file 2 — Figure S1. Plasmid maps [file YEA-36-75-s002.pdf]
