# Supplementary material for: A fast and tuneable auxin‐inducible degron for depletion of target proteins in budding yeast
Source: Yeast. 2018 Nov 12;36(1):75–81. doi: 10.1002/yea.3362 (PMC6587778; doi:10.1002/yea.3362)
Supplement: Supplementary file 3 — Figure S2. Time courses of Yhc1 and Rrp44 depletion with and without addition of auxin. Yhc1 and Rrp44 were AID*‐tagged in strain PZ4EV‐NTIR1 as described in Materials and Methods. β‐estradiol was added at time 0 (T0) either without auxin (left panels) or with auxin addition at a previously determined optimal time (30 min for Yhc1 and 40 min for Rrp44; right panels). Data represent western blot quantifications relative to initial values. [file YEA-36-75-s003.pdf]

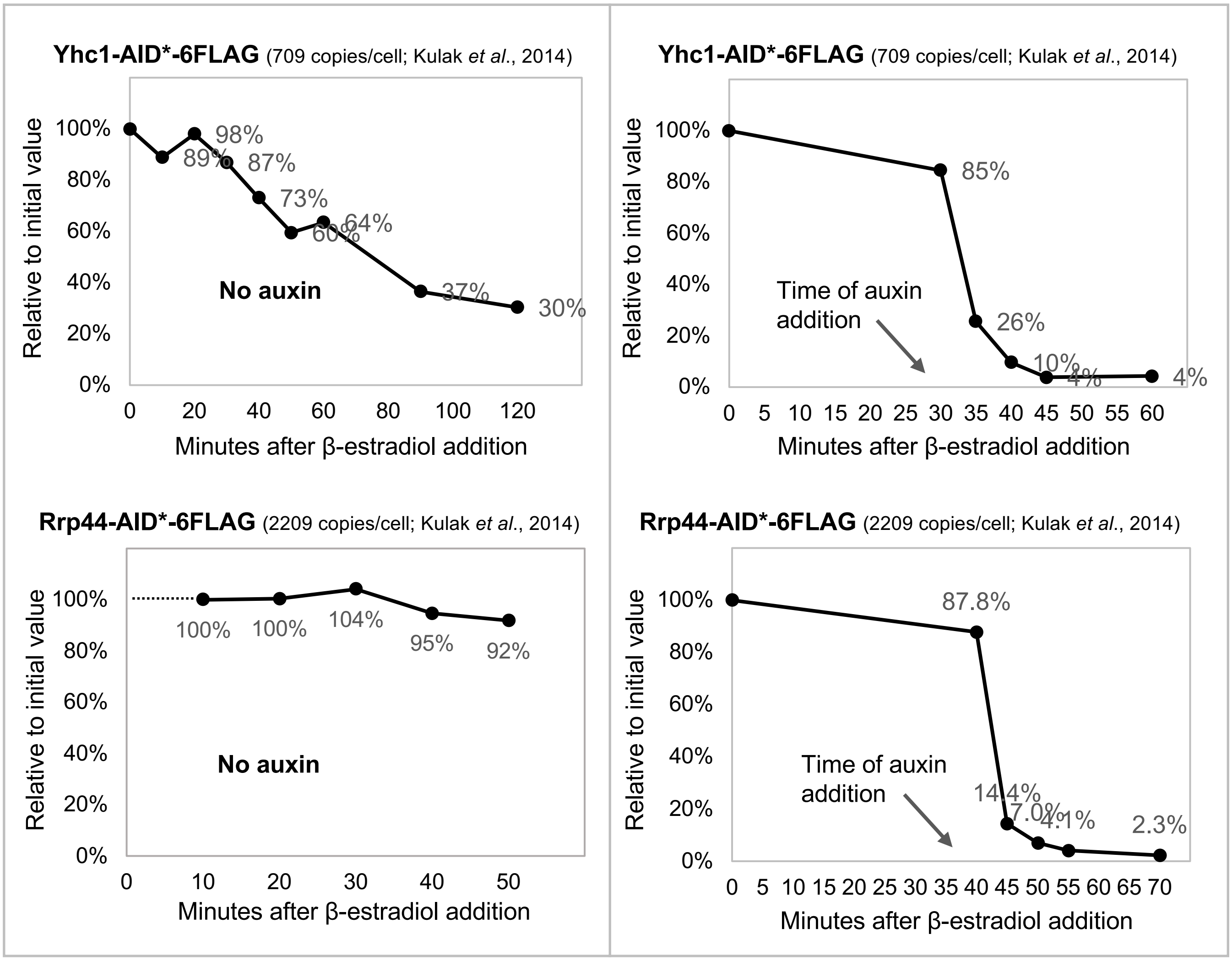

Figure S2. Time courses of Yhc1 and Rrp44 depletion with and without addition of auxin.

Yhc1 and Rrp44 were AID*-tagged in strain PZ4EV-NTIR1 as described in Materials and Methods. β-estradiol was added at time 0 (T0) either without auxin (left panels) or with auxin addition at a previously determined optimal time (30 min for Yhc1 and 40 min for Rrp44; right panels). Data represent western blot quantifications relative to initial values.
